# Supplementary material for: Cargo surface fluidity can reduce inter-motor mechanical interference, promote load-sharing and enhance processivity in teams of molecular motors
Source: PLoS Comput Biol. 2022 Jun 8;18(6):e1010217. doi: 10.1371/journal.pcbi.1010217 (PMC9212169; doi:10.1371/journal.pcbi.1010217)
Supplement: S1 Appendix — (PDF) [file pcbi.1010217.s026.pdf]

Notes on the assumptions in the computational model

1 We neglect the deformation of vesicle due to motor forces.

Application of a point force on the vesicle (the cargo) surface leads to shape deformation which could develop as narrow membrane tubes (tethers) at sufficiently high force magnitudes [2–5]. There have been observations that teams of kinesin motors can pull membrane tubes out of a vesicle [6]. This raises the question as to whether we should consider the alteration in vesicle shape due to tether formation in our computational model. Previous analytical works [2, 3] have shown that the force magnitude needs to be greater than a critical value, $f_c = 2\pi\sqrt{\kappa_c\sigma}$ to pull a nanotube out of a membrane with bending rigidity κ_c and surface tension σ. For typical values, $k_c = 20k_BT = 10^{-19}$ J [5], $\sigma = 10^{-5}$ Nm^{-1} [2, 7] the critical force is $f_c = 8.8$ pN which is higher than the stall force of kinesin (7 pN). This critical value that we computed is lower than the estimated value in at least one other study [2, 6]. We can see from the force distributions in Fig. 2 (b & c) that the typical single motor force values are much lower than this critical value. Hence we have neglected the tether formation in our model.

2 Estimation of rotational diffusion of cargo.

We expect the rotational diffusion to be minimum for a rigid cargo, increase with an increase in cargo surface fluidity and approach the value for free bead in solution at very high cargo surface fluidity. For lipid cargo with high motor diffusivity, one might expect that such rotational diffusion of cargo only renormalizes the diffusion constant of motors on the surface (at most by a factor of 2) but doesn't induce any qualitative change in the motor availability at the access region.

The rotational diffusion constant of a rigid cargo associated with microtubule with a single motor has been measured experimentally to be $D = 7 \times 10^{-2}\,\text{rad}^2 s^{-1}$ for a cargo of about 1.26 μm in diameter [1]. Since this diffusion constant was measured for a cargo of about half-micron radius, if we take into account the possibility that diffusion constant scales as $1/R^3$, for $R = 0.25\mu m$, we may take $D_{0.25\,\mu m} = 1.12\,\text{rad}^2\text{s}^{-1}$ The time required for a cargo to rotate by 90° with this diffusion constant is $\frac{\pi^2}{16D_R} = 0.55s$ which is in the order of the lifetime of single kinesin motor in our simulations (lifetime of kinesin is about 1 s at [ATP] = 2 mM and about 10 s at [ATP] = 4.9 μM). Since how rotational diffusion constant of bead changes as a function of cargo radius is not experimentally measured yet, for our simulations we took rigid cargo diffusion constant D_R to be $7 \times 10^{-2}\text{rad}^2 s^{-1}$ even for $R = 250$ nm (We also used $D_R = 1.12\ \text{rad}^2 s^{-1}$ and compared different metrics like force distribution and off-rate and didn't find considerable difference).

Similarly we can estimate how much is the rotational angular velocity of cargo due to the torque from motor forces. Assume that a microtubule bound motor exerts a torque $\vec{\mathcal{T}}$ on the cargo. Angular velocity of cargo due to this torque is

$$\frac{d\vec{\theta}}{dt} = \frac{\vec{\mathcal{T}}}{8\pi\eta_v R^3} \tag{1}$$

Typical value of tangential component of motor force is $f_{tan} = 1$ pN. So the typical magnitude of the torque on the cargo due to this force is $\mathcal{T} = Rf_{tan} = 2.5 \times 10^{-19}$ Nm. Substituting this in Eq. 1 we get the typical magnitude of angular velocity due to torque from motor forces to be equal to 636 rad s^{-1}. This is a non-negligible angular velocity, for example, the rotation of the cargo due to motor forces in 1 s is 636 rad.

Typical magnitude of the torque, $|\vec{\mathcal{T}}| = R \times f_t$ where f_t is the typical magnitude of tangential force (about 1 pN). So $\frac{d\vec{\theta}}{dt} \propto R^{-2}$. Distance traveled by motor on the cargo surface, arc length $\propto R\frac{d\vec{\theta}}{dt}$. Hence arc length $\propto R^{-1}$. Thus we might expect the rotational distance covered to decrease with increase in cargo radius.

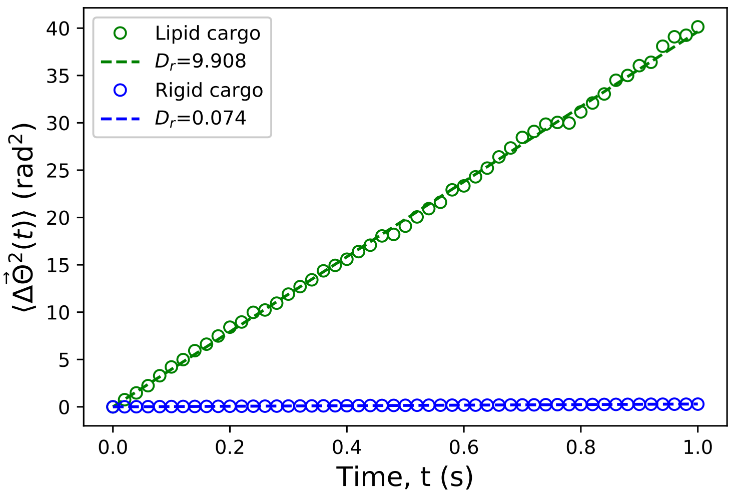

Figure 1: **Benchmark test to verify correct implementation of rotational diffusion.** We ran simulations of cargo dynamics with just rotational diffusion (no motor bound to MT, no transnational diffusion of cargo, no diffusion of motor on cargo surface) and measured the mean-squared-angular-displacement (MSAD) of motor anchor point. Ensemble size = 200. Then we fit MSAD to $4D_R t$ and extracted the rotational diffusion constant, D_R, to verify that we implement the desired rotational diffusion.

Bench mark test and calibration: Mean-Squared-Angular-Displacement

Let A be the anchor position of a motor on the cargo surface and $\hat{a}(t')$ represent the unit vector representing the direction of this anchor position with respect to the cargo center of mass.

Define the angular displacement vector of a motor anchor point as [8]

$$\vec{\Theta}(t) = \sum_{i=0}^{n_t} \Delta\vec{\Theta}(i\Delta t) \tag{2}$$

Where $n_t = t/\Delta t$. The displacement vector $\Delta\vec{\Theta}(t')$ has a magnitude, $|\Delta\vec{\Theta}(t')| = \cos^{-1}\left[\hat{a}(t'-\Delta t)\cdot\hat{a}(t')\right]$ and a direction given by $\hat{a}(t'-\Delta t)\times\hat{a}(t')$. We take $\Delta\vec{\Theta}(0) = 0$.

We then compute the mean squared angular displacement [8]

$$\left\langle \vec{\Delta}\Theta^2(t) \right\rangle = \left\langle \left[\vec{\Delta}\Theta(t) - \vec{\Delta}\Theta(0)\right]^2 \right\rangle \tag{3}$$

As per the Stokes-Einstein-Debye relation we expect the mean squared angular displacement to grow as [8]

$$\left\langle \vec{\Delta}\Theta^2(t) \right\rangle = 4D_R t \tag{4}$$

References

1 Gutiérrez-Medina B, Fehr AN, Block SM. Direct measurements of kinesin torsional properties reveal flexible domains and occasional stalk reversals during stepping. Proc Natl Acad Sci U S A. 2009;106(40):17007–17012. doi:10.1073/pnas.0907133106.

2 Derényi I, Jülicher F, Prost J. Formation and Interaction of Membrane Tubes. Phys Rev Lett. 2002;88:238101. doi:10.1103/PhysRevLett.88.238101.

3 Powers TR, Huber G, Goldstein RE. Fluid-membrane tethers: Minimal surfaces and elastic boundary layers. Phys Rev E. 2002;65:041901. doi:10.1103/PhysRevE.65.041901.

4 Roopa T, Shivashankar GV. Nanomechanics of membrane tubulation and DNA assembly. Appl Phys Lett. 2003;82(10):1631–1633. doi:10.1063/1.1559632.

5 Vutukuri HR, Hoore M, Abaurrea-Velasco C, van Buren L, Dutto A, Auth T, et al. Active particles induce large shape deformations in giant lipid vesicles. Nature. 2020;586(7827):52–56. doi:10.1038/s41586-020-2730-x.

6 Leduc C, Campàs O, Zeldovich KB, Roux A, Jolimaitre P, Bourel-Bonnet L, et al. Cooperative extraction of membrane nanotubes by molecular motors. Proc Natl Acad Sci USA. 2004;101(49):17096–17101. doi:10.1073/pnas.0406598101.

7 Deserno M. Elastic deformation of a fluid membrane upon colloid binding. Phys Rev E. 2004;69:031903. doi:10.1103/PhysRevE.69.031903.

8 Hunter GL, Edmond KV, Elsesser MT, Weeks ER. Tracking rotational diffusion of colloidal clusters Opt. Express 2011;19(18):17189–17202 doi:10.1364/OE.19.017189
