# Supplementary material for: Cargo surface fluidity can reduce inter-motor mechanical interference, promote load-sharing and enhance processivity in teams of molecular motors
Source: PLoS Comput Biol. 2022 Jun 8;18(6):e1010217. doi: 10.1371/journal.pcbi.1010217 (PMC9212169; doi:10.1371/journal.pcbi.1010217)
Supplement: S2 Appendix — (PDF) [file pcbi.1010217.s027.pdf]

Estimation of relevant timescales

1 τ_{bind} and τ_{off}

τ_{bind} is the mean time taken for any one motor out of N motors of lipid cargo to bind to the microtubule. If a single motor takes τ_{ad} to bind, then if we have N of them, mean time for any one of the motor to bind is

$$\tau_{bind} = \frac{\tau_{ad}}{N} \tag{1}$$

$\tau_{ad} = 1/\pi_{ad}$. We have measured the value of π_{ad} in Fig. 3(b) of main text for a lipid cargo of radius 250 nm and fluidity $D = 1\,\mu m^2 s^{-1}$. It is about 0.12 s^{-1}. So $\tau_{ad} = 8.33$ s.

τ_{off} is the time taken for a given bound motor to unbind. It is estimated as $\tau_{off} = \epsilon_o^{-1}$, where ϵ_o is the off-rate of an unloaded kinesin motor (bare off-rate). Unloaded motor off-rate is a function of ATP concentration. At high ATP concentration of 2 mM, ϵ_o is measured to be 0.79 s^{-1} [1,2]. Based on previous studies [3–5], we assume that $\epsilon_o = v_o/d$. d is the run length of unloaded kinesin motor which is found to be independent of ATP concentration. These information along with Michaelis-Menten equation provided in Eq. 16 of main text enable us to calculate unloaded velocity (v_o), unloaded off-rate (ϵ_o) and unbinding time (τ_{off}) of a kinesin-1 motor at different ATP concentrations. Please refer to Table A below for numerical values.

Table A: **Comparison of the single motor binding and unbinding times for different N and [ATP]**

N	[ATP]	v_0 (nm s^{-1})	$\epsilon_o = \frac{v_o}{d}$ (s^{-1})	τ_{off} (s)	$\tau_{bind} = \frac{\tau_{ad}}{N}$ (s)
4	2 mM	800	0.79	1.26	2.08
4	100 μM	555	0.548	1.82	2.08
4	4.9 μM	80	0.079	12.66	2.08
16	2 mM	800	0.79	1.26	0.52
16	100 μM	555	0.548	1.82	0.52

τ_{bind} - the estimated mean time for the first motor binding in a lipid cargo (with $D = 1\ \mu m^2 s^{-1}$) assuming that all motors on the cargo are unbound , τ_{off} - the mean lifetime of a bound motor, N - total motors on the cargo. d is the run length of unloaded kinesin motor which is found to be independent of ATP concentration.

2 Conditional on-rate

In Fig. 3, we showed that the binding rate of a motor increases as the number of bound motors increases. However, it might be challenging to measure this rate directly in experiments because the n-bound state is not stationary but can decay to $n-1$ state by losing a motor. But one might be able to experimentally measure the number of bound motors as a function of time and one can obtain the *conditional on-rate per motor* from this data. We define the conditional on-rate per motor (S10 Fig.) as the rate of transitioning from n-bound state to $n+1$-bound state divided by the total number of unbound motors in that state, $N-n$. A n-bound state can transition to a $n+1$-bound state if any one of the $N-n$ free motors binds to the microtubule. As mentioned earlier n-bound state can also decay to $n-1$-bound state if one of the motors unbind. So we are looking at the rate with which n goes to $n+1$ gated by n going to $n-1$. To obtain this conditional on-rate from the number of bound motors as a function of time, we just have to filter all the n to $n+1$ transitions in this time series, measure mean rate for such transitions and divide by $N-n$.

In addition to being straightforward to measure from trajectory data, it is also easy to find an analytical estimation of this conditional on-rate of a motor if we know the single motor on-rate and the single motor off-rate. Let τ_o^n be the mean time for the decay of the n-bound state. Assuming that the motors work independently, we can write, $\tau_o^n = \tau_o^1/n$. Let τ_b^n be the mean time for gaining 1 more bound motor. Since there will be $N-n$ free motors when n motors are bound and these motors bind independently, $\tau_b^n = \tau_b^s/(N-n)$ where $\tau_b^s = 1/\pi_{ad}$ is the binding time of a single motor.

Mean time to go from n state to $n+1$ state before n state decays to $n-1$ state is

$$\tau_{cd}^n = \frac{1}{\mathcal{N}} \int_0^\infty t e^{-t/\tau_m} \frac{1}{\tau_b^n} dt \tag{2}$$

where

$$\frac{1}{\tau_m} = \frac{1}{\tau_o^n} + \frac{1}{\tau_b^n}$$

$\mathcal{N}$ is the normalization factor given by

$$\mathcal{N} = \int_0^\infty e^{-t/\tau_m} \frac{1}{\tau_b^n} dt \tag{3}$$

On simplification

$$\tau_{cd}^n = \tau_m = \frac{\tau_b^n \tau_o^n}{\tau_b^n + \tau_o^n} \tag{4}$$

The conditional binding time per motor is $\tau_{cd}^1 = \tau_{cd}^n(N-n)$. Conditional on-rate per motor is $\pi_{cd} = 1/\tau_{cd}^1$.

We note that as n increases, the decay rate of n-bound state also increases. This implies the conditional on-rate per motor should increase as a function of the number of bound motors, just because of the increase in the decay rate (or decrease in the gating time) even if the binding rate of motor π_{ad} doesn't change.

But we have seen in Fig. 3(b) that π_{ad} is not a constant value but is a function of n. We were curious to know how much difference will the change in π_{ad} make on the conditional on-rate as a function of n. So we plotted the estimated conditional on-rate taking $\pi_{ad}(n)$ (data from Fig. 3(b)) and compared with conditional on-rate with π_{ad} a constant (equal to $\pi_{ad}(1)$) along with the measured value from simulations (S10 Fig.). However, there is not much difference between $\pi_{ad}(n)$ and $\pi_{ad}(1)$ case indicating that it might be challenging to observe this in experiments. The conditional on-rate measured from simulations agree well with the analytical estimations confirming the consistency between simulation measurements and analytical calculations.

References

1 Arpag G, Shastry S, Hancock WO, Tüzel E (2014) Transport by populations of fast and slow kinesins uncovers novel family-dependent motor characteristics important for in vivo function. *Biophys. J.* 107(8):1896–1904.

2 Andreasson JOL (2013) Ph.D. thesis (Stanford University, Stanford,CA).

3 Xu J, Shu Z, King SJ, Gross SP (2012) Tuning Multiple Motor Travel via Single Motor Velocity. *Traffic* 13(9):1198–1205.

4 Yajima J, Alonso MC, Cross RA, Toyoshima YY (2002) Direct Long-Term Observation of Kinesin Processivity at Low Load. *Curr. Biol.* 12(4):301–306.

5 Seitz A, Surrey T (2006) Processive movement of single kinesins on crowded microtubules visualized using quantum dots. *EMBO J.* 25(2):267–277.
