# Supplementary material for: Cargo surface fluidity can reduce inter-motor mechanical interference, promote load-sharing and enhance processivity in teams of molecular motors
Source: PLoS Comput Biol. 2022 Jun 8;18(6):e1010217. doi: 10.1371/journal.pcbi.1010217 (PMC9212169; doi:10.1371/journal.pcbi.1010217)
Supplement: S3 Appendix — (PDF) [file pcbi.1010217.s028.pdf]

Analytical estimation of run length for a rigid cargo

Since motors, are not free to move on a rigid cargo we approximate the run length as a weighted average of run lengths for the different number of motors in the access region [1]. The probability of locating exactly l motors in the access region when N motors are present on the cargo is

$$P(l) = \binom{N}{l} \alpha^l (1-\alpha)^{N-l} \tag{1}$$

α is the ratio of the access region to the total surface area of the cargo. The run length of the rigid cargo is estimated as

$$r_{rigid} = \sum_{l=1}^{N} \frac{P(l)}{\mathcal{N}} \, r(l, \pi_o) \tag{2}$$

where $\mathcal{N} = \sum_{l=1}^{N} P(l)$ and $r(N_a, \pi_{ad})$ is given by [2]

$$r(N_a, \pi_{ad}) = \frac{v_o}{N_a \pi_{ad}} \left[\left(1 + \frac{\pi_{ad}}{\epsilon_m} \right)^{N_a} - 1 \right] \tag{3}$$

References

1 Li Q, King SJ, Gopinathan A, Xu J (2016) Quantitative Determination of the Probability of Multiple-Motor Transport in Bead-Based Assays. *Biophys. J.* 110(12):2720–2728.

2 Klumpp S, Lipowsky R. Cooperative Cargo Transport by Several Molecular Motors. Proc Natl Acad Sci USA. 2005;102(48):17284–17289.
