# Supplementary material for: Cargo surface fluidity can reduce inter-motor mechanical interference, promote load-sharing and enhance processivity in teams of molecular motors
Source: PLoS Comput Biol. 2022 Jun 8;18(6):e1010217. doi: 10.1371/journal.pcbi.1010217 (PMC9212169; doi:10.1371/journal.pcbi.1010217)
Supplement: S4 Appendix — (PDF) [file pcbi.1010217.s029.pdf]

Cargo runlength can be different even though the average number of motors are the same

We observe in Fig. 5(a) that the average number of motors for N = 4 Low ATP (4.9 μM) and N = 16 High ATP (2 mM) are approximately equal for lipid cargoes ($D = 1$ μm^{2}s^{-1}). However, the runlength for the former case is higher than the latter. This is surprising since intuitively one would expect runlength to be the same when the average number of motors is the same.

We attribute this difference to the higher tendency to accumulate motors in the case of N = 4 Low ATP than N = 16 High ATP. A cargo run stops when the last bound motor unbinds. The likelihood for a new motor to bind before this last motor unbinds determines how far the cargo travels on the MT. Let us look at the ratio of the rate to bind one more motor to the unbinding rate for the case when there is just one motor bound, i.e.,

$$\mathcal{R} = \frac{1 \longrightarrow 2}{1 \longrightarrow 0} \tag{1}$$

Calculated value of effective binding rate for a given motor in lipid cargo with $D = 1$ μm^{2}s^{-1} is approximately 0.12 s^{-1}. So the rate for the process $1 \longrightarrow 2$ is 0.12(N-1) s^{-1}. The approximate unbinding rate, i.e., the rate $1 \longrightarrow 0$ is 0.79 s^{-1} for High ATP and 0.079 s^{-1} for Low ATP (The no load off-rate of a single kinesin motor).

(a) For N = 4 Low ATP (4.9 μM)

$$\mathcal{R}_1 = \frac{(4-1) * 0.12}{0.079} = 4.56 \tag{2}$$

(b) For N = 16 High ATP (2 mM)

$$\mathcal{R}_2 = \frac{(16-1) * 0.12}{0.79} = 2.28 \tag{3}$$

So the accumulation tendency in case (a) is higher than in case (b). Assume during a cargo run, the bound motor number becomes 1, then case (b) is more likely to fall off compared to case (a). However the average number of bound motors turns out to be the same because of the difference in upper bound, the maximum number of bound motors possible in case (a) is 4 whereas in case (b) it is 16.
