# Supplementary material for: Cargo surface fluidity can reduce inter-motor mechanical interference, promote load-sharing and enhance processivity in teams of molecular motors
Source: PLoS Comput Biol. 2022 Jun 8;18(6):e1010217. doi: 10.1371/journal.pcbi.1010217 (PMC9212169; doi:10.1371/journal.pcbi.1010217)
Supplement: S5 Appendix — (PDF) [file pcbi.1010217.s030.pdf]

1 Details of the metrics used in Fig. 2

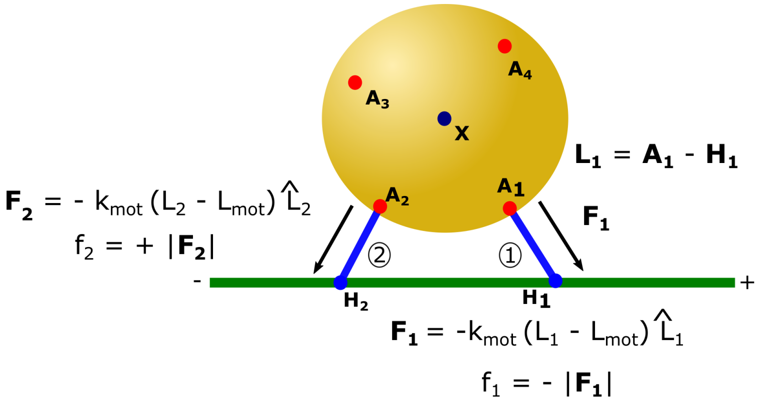

Figure 1: **Schematic with the motor forces** $\vec{X}$ is the center of mass of the cargo. $\vec{A}_i$ are the anchor position vectors of motors. $\vec{H}_i$ are the position vectors of the microtubule-bound motor heads. Corresponding motor forces are represented by $\vec{F}_i$.

Force distributions

As explained in the main text, Fig. 2 (b and c) show the distributions of the magnitude of individual motor forces. To get this data, we first run simulations of cargo transport for different cargo surface fludities (N=16, [ATP]= 2 mM). For each cargo run, we recorded data samples at every 0.01 s intervals. Every time sample includes the position of center of mass ($\vec{X}$), the anchor positions of all motors ($\vec{A}_i$), the head positions of microtubule bound motors ($\vec{H}_i$). To get good statistics we ran 200 cargo runs for each cargo surface fluidity.

From the collected data set, we extract all those time samples with desired number of bound motors (say $n = 3$). Then we compute the motor force vectors ($\vec{F}_i$) from the motor anchor and head positions as described in the materials and methods (Also see Fig. 1). Then we compute the magnitude of the force vectors ($f_i = |\vec{F}_i|$). As per the convention, we take the magnitude to be negative for motors experiencing a hindering load (for example, motor 1 experiences hindering load in the Fig. 1) and positive for motors experiencing assistive load (motor 2 in Fig. 1). We randomly select 10000 such f values from all time samples with $n = 3$ and then we compute the force distribution and the error bars by bootstrapping method. Bootstrap sample size was 1000 and number of bootstrap samples was 10.

Mean variance of forces

For each time sample with the given number of bound motors, we computed the variance of the absolute magnitude of motor forces $\sigma^2_{|f|} = \frac{1}{n-1}\sum_{i=1}^{n}(f_i - \bar{f})^2$ where $\bar{f} = \frac{1}{n}\sum_{i=1}^{n} f_i$. We then computed the mean of this variance by using S=10000 random time samples, $\frac{1}{S}\sum_{i=1}^{S}\sigma^2_{|f|}(i)$.

Force correlation

At every time sample with a given number of bound motors (n), we computed the product of x-components, $f_x^j f_x^k$, ($j \neq k$) between all different unique pairs (i, j) of bound motors. Then we computed the mean by randomly drawing $S = 10000$ values from the set of all $f_x^j f_x^k$ values over all independent motor pairs in all the time samples with given number of bound motors from 200 cargo runs.

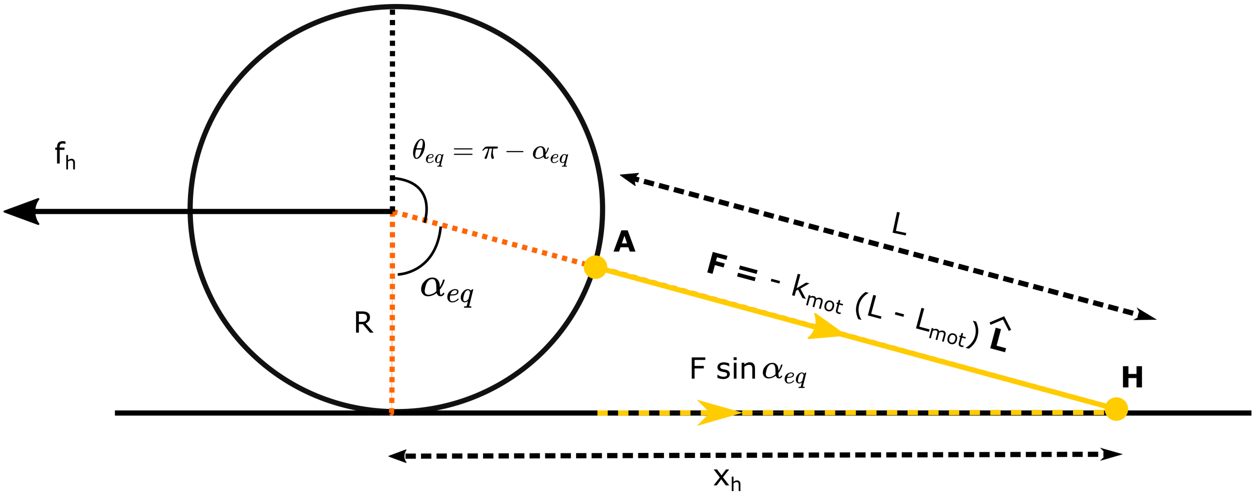

Figure 2: **Schematic diagram of a cargo with 1 motor under a fixed hindering load.** It is assumed that motor diffuses until the tangential component of force on the cargo surface reduces to 0.

Motor off-rate

We measure the motor off-rate by tracking each individual motor from the time binds to the microtubule to the time it detaches from the microtubule. By collecting lifetime of many such individual motor runs within a cargo run, we compute the mean lifetime of motors.

2 Calculation of equilibrium anchor position for a motor on a lipid cargo (for Fig 6)

We assume that motor diffuses on the cargo until the tangential component motor force reduces to zero and at equillibrium, x-component of motor force balances the hindering load. Thus from force balance,we have

$$F \sin \alpha_{eq} = f_h \tag{1}$$

$$F = k_{mot}(L - L_{mot}) \tag{2}$$

But

$$L = \frac{x_h}{\sin \alpha_{eq}} - R = \frac{R}{\cos \alpha_{eq}} - R \tag{3}$$

Hence,

$$F = k_{mot} \left(\frac{R}{\cos \alpha_{eq}} - R - L_{mot} \right) \tag{4}$$

Substituting Eq. 4 in Eq. 1, we get

$$f_h = k_{mot} \sin \alpha_{eq} \left(\frac{R}{\cos \alpha_{eq}} - R - L_{mot} \right) \tag{5}$$

We then solved for $cos\alpha_{eq}$ numerically. We found solutions range from $cos\, \alpha_{eq} = 0.81$ to 0.72 as hindering force varies from 0 to 8 pN. This is comparable to the value $cos\, \alpha$ for locations of motor accumulation we get from simulations (0.98, 0.83, 0.83 for $f_h = 0$, 2, 4 pN respectively). The predicted values from the force equilibrium are at slightly larger angles than the measured simulation values presumably because fluctuations will tend to push the cargo slightly away from the MT.
