# Supplementary material for: Cargo surface fluidity can reduce inter-motor mechanical interference, promote load-sharing and enhance processivity in teams of molecular motors
Source: PLoS Comput Biol. 2022 Jun 8;18(6):e1010217. doi: 10.1371/journal.pcbi.1010217 (PMC9212169; doi:10.1371/journal.pcbi.1010217)
Supplement: Attachment — Submitted filename: Gopinathan-Response.pdf [file pcbi.1010217.s031.pdf]

The manuscript by Sarpangala and Gopinathan builds on earlier work to understand how multiple motors cooperate/not cooperate on a cargo as they transport it along microtubules. The advancement here is a computational analysis of how the cargo membrane allows the cargo-motor attachment to diffuse around. How might this diffusion relieve strain on a motor as it is generating force, leading to change in the performance of a motor (and therefore groups of motors). This is a very relevant topic at this juncture of our understanding of molecular motors, so I support publication. I appreciate the thorough analysis and the attempt to relate to experimental results throughout the manuscript. However, there are several issues that should be addressed/clarified before the paper can be published.

MAJOR COMMENTS

1) The authors appear to take a very one-sided (positive) view of the effect of diffusion on motor-groups. While diffusion can prevent negative interference as described here, it can also be a problem for a motor that "slips" when it is trying to generate force. If that happens, motor performance can be compromised. Infact, it has been suggested that the presence of motors (both dynein and kinesin) in ordered regions of the membrane can improve performance by providing a rigid substrate against which the motor can generate force:-

http://www.cell.com/cell/abstract/S0092-8674(15)01718-3
… for what might happen on a real cellular cargo
Grover et al Ref 38 in the manuscript.
Klopfenstein et al Cell, Vol. 109, 347–358, May 3, 2002

Why is this issue being completely ignored? A possible resolution to this caveat is that as it tries to generate force (on a spherical cargo), the motor can diffuse on the membrane and come to a point where the force-vector is along the radial direction (no tangetial force, so no more slipping). I recommend that this question be addressed using their simulations and a section be added accordingly. The relevant literature (above two papers and others) should also be discussed. The authors should also look up the below listed papers in this context :-

https://www.pnas.org/content/pnas/101/49/17096.full.pdf
doi: 10.1529/biophysj.107.118554

2) The results presented here depend on computation. Therefore, the following claims in abstract should be tempered down/removed.

"We showed explicitly, for the first time, that surface fluidity leads to the reduction of negative mechanical interference… ; Remarkably, we also showed that, independent …"

You have not **really** showed that this is true, you have only shown this on a computer. Infact, the "Author Summary" provides a much more balanced and acceptable perspective

3) The Introduction is completely missing a discussion on the extensive work done using purified cellular organelles (that have a membrane containing cholesterol). Is this so because those papers present a different picture from what the authors want to convey here? For example, see :-

http://www.cell.com/cell/abstract/S0092-8674(15)01718-3
Klopfenstein et al Cell, Vol. 109, 347–358, May 3, 2002 (Check Fig 3 in that paper)

MINOR COMMENTS

"We expected that the motor attachment point's freedom to move on the lipid cargo surface in response to forces could relieve strain and reduce the forces experienced by the motor."
This mention of "forces" is confusing at the beginning of the paper – it can be confused with the intrinsic force generated by kinesin. Explain here how this force is calculated in simple words, and what exactly it means - add an equation here if needed (no need to hide all the equations in the methods section !!).

I think Fig S1 shows a very important result – distribution of forces in hindering and assistive direction. This should move to the main paper. Can you also attach a small schematic inside this panel to explain what hindering and assistive means??

Fig S2- the threshold of 0.01Fs for "high force" seems arbitrary. This threshold (1% of Fs) also appears very low to me. For example, such a low force cant even be measured in an optical trap experiment (due to noise). This threshold needs revision (or a suitable justification)

The effects in Fig 2f and g appear minor -- move f and g to supp figs?? Also, (2G) the really small increase in on rate -- what does it mean for processivity of n motors? Must have this explanation, otherwise this result is just "hanging around"

"For a cargo bound to the MT with one or more motors, this access area is a function of the cargo geometry and the proximity of the cargo to the MT"
The following paper should be cited in this context (not just because it is our paper), but because it is perhaps the first experimental paper where this access area has been calculated and used to understand real experimental data.
http://www.cell.com/cell/abstract/S0092-8674(15)01718-3

Fig 3B :- Hard to understand with all the colour coding. Can you explain the relevance of polar angle by using a schematic fig here?

Fig 3C:- For rigid at N=16, what is the number of motors that can access microtubule? Must be very low because 1.6 micron run length is very low for a cargo with such a large number of kinesins. This point needs clarification because it appears to contradict all the long bead movement seen in vitro with high density of purified kinesin.
