# Supplementary material for: Cargo surface fluidity can reduce inter-motor mechanical interference, promote load-sharing and enhance processivity in teams of molecular motors
Source: PLoS Comput Biol. 2022 Jun 8;18(6):e1010217. doi: 10.1371/journal.pcbi.1010217 (PMC9212169; doi:10.1371/journal.pcbi.1010217)
Supplement: Attachment — Submitted filename: response letter.pdf [file pcbi.1010217.s032.pdf]

We appreciate the very careful and thorough reading of the manuscript by the reviewers and the very helpful and detailed constructive criticisms and suggestions. Based on the comments and suggestions, we have made major changes to the paper that have overall tremendously improved the quality, readability and potential impact. We are very much indebted to the referees for their time and effort! We have included them in our acknowledgments (if that is ok with them) as a token of our appreciation.
We also apologize for the long turnaround time, but as you will see the changes and additions we have made are quite substantial. In brief, we included rotations into our simulations and re-did most of the presented results. We also looked at motor-team clustering and collective force generation under load adding two entirely new sections. Below, we address the reviewer's comments (in blue) point by point. We note that changes to the manuscript are indicated in blue text in the main paper draft.

Reviewer #1:

The manuscript by Sarpangala and Gopinathan builds on earlier work to understand how multiple motors cooperate/not cooperate on a cargo as they transport it along microtubules. The advancement here is a computational analysis of how the cargo membrane allows the cargo-motor attachment to diffuse around. How might this diffusion relieve strain on a motor as it is generating force, leading to change in the performance of a motor (and therefore groups of motors). This is a very relevant topic at this juncture of our understanding of molecular motors, so I support publication. I appreciate the thorough analysis and the attempt to relate to experimental results throughout the manuscript. However, there are several issues that should be addressed/clarified before the paper can be published.

We appreciate the reviewer's kind words regarding relevance and effort.

MAJOR COMMENTS 1) The authors appear to take a very one-sided (positive) view of the effect of diffusion on motor groups. While diffusion can prevent negative interference as described here, it can also be a problem for a motor that "slips" when it is trying to generate force. If that happens, motor performance can be compromised. Infact, it has been suggested that the presence of motors (both dynein and kinesin) in ordered regions of the membrane can improve performance by providing a rigid substrate against which the motor can generate force:- http://www.cell.com/cell/abstract/S0092-8674(15)01718-3 … for what might happen on a real cellular cargo Grover et al Ref 38 in the manuscript. Klopfenstein et al Cell, Vol. 109, 347–358, May 3, 2002 Why is this issue being completely ignored?
A possible resolution to this caveat is that as it tries to generate force (on a spherical cargo), the motor can diffuse on the membrane and come to a point where the force-vector is along the radial direction (no tangential force, so no more slipping). I recommend that this question be addressed using their simulations and a section be added accordingly.

The relevant literature (above two papers and others) should also be discussed. The authors should also look up the below listed papers in this context :-
https://www.pnas.org/content/pnas/101/49/17096.full.pdf doi: 10.1529/biophysj.107.118554

We appreciate this excellent point from the reviewer. It is certainly an aspect that should have been addressed. We have therefore added a whole new subsection in the results section titled *"Motors dynamically cluster at specific locations on the lipid cargoes"* where we

address this concern. For this section, we performed simulations with different hindering loads on the cargo and observed that motors in lipid cargo slide and accumulate at specific locations on the cargo - exactly as suggested by the referee. This clustering location was found to be close to where we would expect the motor force to be completely in the radial direction. This adaptive clustering of the motors producing a directed, collective force may have other implications like deformation and membrane tube extraction that we also discuss. We have related and discussed our results in the context of the references kindly pointed out to us.

2) The results presented here depend on computation. Therefore, the following claims in abstract should be tempered down/removed. “We showed explicitly, for the first time, that surface fluidity leads to the reduction of negative mechanical interference… ; Remarkably, we also showed that, independent …” You have not really showed that this is true, you have only shown this on a computer. Infact, the “Author Summary” provides a much more balanced and acceptable perspective

We agree that our enthusiasm might be misinterpreted. Wherever appropriate, we have replaced most of the “we show” with “we predict” or “simulations show”.

3) The Introduction is completely missing a discussion on the extensive work done using purified cellular organelles (that have a membrane containing cholesterol). Is this so because those papers present a different picture from what the authors want to convey here? For example, see :- http://www.cell.com/cell/abstract/S0092-8674(15)01718-3 Klopfenstein et al Cell, Vol. 109, 347–358, May 3, 2002 (Check Fig 3 in that paper)

We thank the referee again for pointing out these relevant studies. While we do not study the effects of clustered motors *per se*, we have discussed this as a possible mechanism in the introduction as well as in the results subsection *“Motors dynamically cluster at specific locations on the lipid cargoes”* where we discuss the dynamic clustering we observe under load as distinct from membrane micro-domain induced clustering.

MINOR COMMENTS

“We expected that the motor attachment point’s freedom to move on the lipid cargo surface in response to forces could relieve strain and reduce the forces experienced by the motor.” This mention of “forces” is confusing at the beginning of the paper – it can be confused with the intrinsic force generated by kinesin. Explain here how this force is calculated in simple words, and what exactly it means - add an equation here if needed (no need to hide all the equations in the methods section !!).

We appreciate this input and agree we could be clearer here. We have now added a schematic (Fig 2a) that shows the motor forces, how we compute them, and also our sign convention for motor force magnitudes under hindering and assisting loads. Hopefully this illustrates our procedure better.

I think Fig S1 shows a very important result – distribution of forces in hindering and assistive direction. This should move to the main paper. Can you also attach a small schematic inside this panel to explain what hindering and assistive means??

We thank the reviewer for appreciating the importance of this result. We have replotted this data for easier visualization and also added small schematics inside the figure that show motors experiencing hindering and assisting loads (Fig 2c**)**.

Fig S2- the threshold of 0.01Fs for "high force" seems arbitrary. This threshold (1% of Fs) also appears very low to me. For example, such a low force can't even be measured in an optical trap experiment (due to noise). This threshold needs revision (or a suitable justification)

We agree that the reviewer is indeed correct that 0.01Fs =0.07 pN is indeed a very low value to be termed a threshold for "high force". We actually meant to refer to forces that were "not negligibly small". We have therefore dropped the use of the term "high force" and replaced it with "non-negligible". This threshold, however, does serve the purpose of showing how heavy the tail of the distribution is. Since, the y-axis of the distribution in Fig 2 (b,c) is logarithmic, there is a large change in the probability of forces experienced by motors with very small change in forces at values close to zero. So the cut off (>0.01Fs) essentially shows the fraction of time motors experience non-negligible forces. So we continue to use same threshold value but refer to it more accurately as "non-negligible" force..

The effects in Fig 2f and g appear minor -- move f and g to supp figs?? Also, (2G) the really small increase in on rate -- what does it mean for processivity of n motors? Must have this explanation, otherwise this result is just "hanging around"

We agree that the change in value appears to be minor because cargo height changes by only 10 nm as the number of bound motors changes from 1 to 5, *however* the corresponding change in on-rate is about 33% (0.12 to 0.16) (see new Fig 3 a,b). This change in on-rate *does* influence the overall processivity of teams. In fact, we make analytical estimates of how much of the change in the runlength of lipid cargo is due to this effect (see Fig 5c). We have now added a sentence directing the readers to this analysis in the last but one paragraph of the results subsection *"On-rate of a motor increases with the number of bound motors for fluid cargo"*.

"For a cargo bound to the MT with one or more motors, this access area is a function of the cargo geometry and the proximity of the cargo to the MT" The following paper should be cited in this context (not just because it is our paper), but because it is perhaps the first experimental paper where this access area has been calculated and used to understand real experimental data. http://www.cell.com/cell/abstract/S0092-8674(15)01718-3

We apologize for having missed this study. It is indeed one of the first papers that discusses this effect. Now we have cited this paper in the first two paragraphs of our results subsection *"On-rate of a motor increases with the number of bound motors for fluid cargo"*

Fig 3B :- Hard to understand with all the colour coding. Can you explain the relevance of polar angle by using a schematic fig here?

We have now added a schematic to show the polar angle. Please find below the revised figure which is now Fig 4.

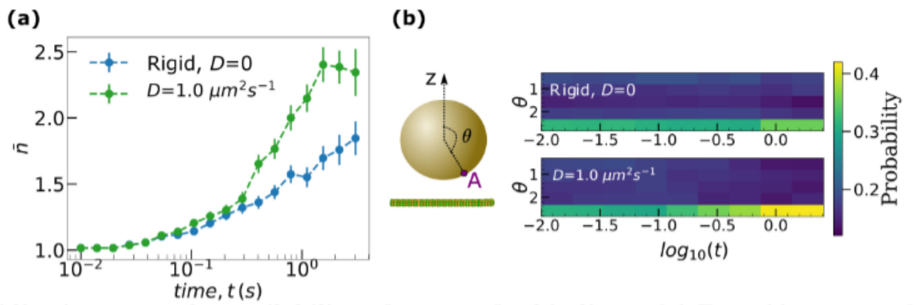

Fig 3C:- For rigid at N=16, what is the number of motors that can access microtubule? Must be very low because 1.6 micron run length is very low for a cargo with such a large number of kinesins. This point needs clarification because it appears to contradict all the long bead movement seen in vitro with high density of purified kinesin.

As the reviewer rightly deduces, this number is indeed very low for rigid N=16. The estimated number of motors that can access the microtubule is about 1.37. A cargo can bind to the microtubule only when there is at least 1 motor in the access area. So we estimate this average number of motors that can access the microtubule as N_a = 1 + (S_a / S_T) = 1.37. S_a = 1.913238e-14 m^2 is the computed access area for a height of cargo equal to the average height of cargo when a single motor is attached. We compared our runlength values with other similar models and also experiments (Bovyn et. al. 2021, Jiang et. al. 2019, and Li et. al. 2018) and found good agreement. We added this discussion in the subsection *"Fluid cargo have longer run lengths than rigid cargo"*.

Reviewer #2:

In this paper Sarpangala and Gopinathan present simulations of a Brownian Dynamics model of transport of fluid and rigid cargo by multiple Kinesin-I motors. They find that membrane fluidity decreases negative interference between multiple bound kinesin motors, and increases motor binding (under some conditions). Additionally, they find that, regardless of membrane fluidity, Kinesin binding to the microtubule increases the binding of subsequent Kinesin by pulling the cargo closer to the microtubule. They do some analysis to estimate the

contribution of these effects, and determine that the decrease in negative interference is the dominant effect under typical in vivo conditions.

For the most part, the model seems reasonable (though I did have some questions about it, see below). I particularly liked the use of both simulation and analysis to address questions of interest. Although the paper would be stronger with experimental support of its predictions, similar models have been shown to be sufficiently predictive that I thought the results could stand on their own. I found the results interesting, however I was left with a few major concerns and questions.

First, how does this model differ from previous models, and what unique insights does it provide? Second, I found the paper difficult to read because I could not understand the results until I knew what went in to the model. Third, though most of the modeling seemed reasonable, I was not convinced that cargo rotation could be reasonably ignored. It is the first of these concerns that, to me, is most important. I will explain each concern more fully, and then follow with some more minor concerns.

Concern 1: How does this model differ from previous models?

Overall, I felt the paper was too focused on the results of the modeling, and not enough on how the results fit into previous modeling work.

One major concern I had reading the paper is that the model seems very similar to the model presented in Bovyn et al. 2021. To be fair, this paper is cited (reference 36 of the main text). However, the similarities and differences between that model and the authors' model was not discussed, nor were the similarities and differences between the results of that study and authors' results. In my mind, that's the purpose, in part, of a Discussion — i.e., to discuss how the current results relate to the literature.

Indeed, as the reviewer points out, our model is quite similar to that of Bovyn et. al. We have now described the differences and similarities between our models in the introduction (second paragraph). While the models are very similar, the focus of our paper is different, looking at the effects of fluidity at the motor level including interference between motors and also studying ATP dependence and collective force generation. Not surprisingly, given the similarity of models, many of the measurements of the same quantities such as run lengths, mean time for motors to bind etc. are consistent. We now discuss these consistencies in the appropriate results sections.

Building off this, although the authors do cite a good selection of modeling literature, it is incomplete and misses some recent studies that consider transport by multiple kinesin motors (e.g. Chen, Nam, Epureanu, PRE, 2018 and 2020; Takshak, Mishra, Kulkarni and Kunwar, Physica A, 2017) multiple dynein motors (Chowdhary, Kaplan, Che, Cui, Biophys J, 2018) and multiple myosin motors (Lombardo et al., Nature Comm 2017, PNAS 2019). Some of these consider fluid liposomes (e.g. Chowdhary and Lombardo). Note that

these are just references that I can think of now, and not an exhaustive list. As with Bovyn et al, the authors' model should, I think, be contrasted with these and the results compared.

We thank the reviewer for these excellent references and we have included them in our discussions of our model especially in the *Materials and Methods* section and also compared with some of our results as appropriate.

As an example of a comparison that should, I think, be made is the author's result of positive cooperativity — i.e., the increasing binding rate that occurs when kinesin motors bind — with negative cooperativity discussed in Lombardo et al. 2017. In that paper, the authors' model predicts that as myosin motors bind to actin, they restrict the movement of the cargo which then decreases the binding rate of subsequent motors. Moreover, they perform measurements in the optical trap to support this prediction. I believe the difference arises from different mechanics of the motors. In particular, kinesin are rather floppy while myosin are stiffer — however it might also arise from the fact that the authors neglect rotation of the cargo, and don't include a torsional spring element that tends to keep the kinesin motors extending from the cargo in the normal direction.

This is an excellent point and we thank the reviewer for sharing. We actually have now included rotations (see concern 3 for details) and the effect is still there. While stiffness/compressibility may be a factor, a more important factor might be that we do not have competition for binding on the MT (because of multiple protofilaments). In myosin/actin, competition for binding sites will be a limiting factor and this was correctly included in the Lombardo et al model leading to negative cooperativity. We have included this discussion in the "*Results & Discussion*" (subsection "*On-rate of a motor increases with the number of bound motors for fluid cargo*")

Concern 2: The results are hard to understand without a description of the model.

While I think it's ok to leave the details of the model to a methods section at the end of the paper, there must be some description of the model prior to the results. Without knowing the details of the mechanochemical cycle each kinesin undergoes (including how ATP fits in), the mechanical model of the kinesin molecules and other such details, it is impossible to understand where the results come from. As a particular example, the result of positive cooperativity arises from the assumption in the model that kinesin can bind to the microtubule if it is within a distance L_mot of the kinesin's attachment to the cargo. As mentioned above, different assumptions about the mechanics of this connection can lead to negative cooperativity (e.g. Lombardo et al. 2017). Understanding how motors working together might increase velocity depends, at least in part, on the mechanochemical model of the motors (e.g. Nelson et al. 2014, PNAS, reference 42). Finally, I didn't realize that cargo rotation was neglected in the model until I read the supplement.

I suggest that the model be described at the beginning of the Results. The precise details of the modeling and the equations might, perhaps, be saved for the methods section; however, there must be enough detail for the reader to understand and evaluate the rest of the Results section.

We agree with the reviewer that not sufficient information was presented before moving to the results. We have now added more details of the model in the *"Introduction"* section. We have included the details of the mechanical model of kinesin and how ATP fits in and also the assumption that kinesin can bind with specific rate if it is within a distance of L_mot from the microtubule. We also have a new schematic (Fig 1c) which will hopefully help with a better preliminary understanding of the model.

Concern 3:

I do not understand and am therefore not convinced by the authors arguments that rotational motion of the cargo may be neglected (see below):

In my reading of Gutierriez-Medina et al., it seemed to me that they measured a rotational diffusion constant of 7*10^-2 rad^2/s, a bit over an order of magnitude larger than that reported in Appendix S1. With this value, the time to rotate 90 degrees is 1 second, much more comparable to the stepping rate.

In addition, they find that double-headed kinesin applies a significant torque to the bead, which restrains its motion. This is not included in the model. In the same appendix, if I assume that the applied torque scales as f*R, I get 2.5*10^-19 Nm (1*10^-12*250*10^-9), in agreement with the authors. However, when I divide this value by 8*pi*eta_v*R^3 — which I calculate to be 8*pi*0.001*Pa*s*(250*10^-9 m)^3 = 3.8*10^-22 N*m*s, I get a rotational diffusion constant of order 10^4 — fifteen orders of magnitude larger than the value reported in Appendix S1. Intuitively, this makes some sense, since I would expect the bead to rotate as the motors step — indeed, such rotation was measured by Gutierrez-Medina et al, referenced above.

We are indebted to the reviewer for taking the time to check this calculation. We agree with the reviewer on both of these points. We made an arithmetical error while calculating the amount of rotation previously. We have redone the calculation (S1 Appendix) and consistent with the reviewer we find that, while rotation is not large, it is not small either. Thus it was now not clear that rotation could be safely ignored. We therefore took the plunge and **incorporated rotation into our model**. This was a major effort and we have updated most of our result figures to include rotations. We analyzed the impact this rotation has on the several metrics we measured, like the force

correlation, motor off rate , cargo height etc. We describe in detail our method (*"Materials and Methods"* section) and contrast results with and without rotations both in the main paper and in supporting information.

Briefly, we did find that rotation influences the amount of negative interference and run length. Motors experience higher forces and hence higher off-rates with rotation. The height of the cargo above the MT is observed to be reduced when there is rotation. Finally the run length remains about the same (with perhaps a slight decrease of less than 10%) when we introduce rotation. However, there is no qualitative change and only a minor quantitative change in how the metrics compare between rigid and lipid cargo, which means that the introduction of rotation does not influence any of the conclusions we arrived at in the paper.

It should be noted that, rather than introduce rotations as an add-on after presenting the model in the absence of rotations, for the ease of readability and coherent development for the new reader, we introduce the full model and then discuss the differences in the absence of rotations as an interesting aspect.

My understanding of Bovyn et al. 2021, is that they included rotational diffusion in their model, and found it important in their subsequent results.

Yes, the reviewer is correct that Bovyn et. al. included rotations and found that rotation is important in their results but only specifically in the case of initial binding rate. However, it is unclear how much influence rotation has on the other metrics they measured such as the runlength and number of engaged motors. They do claim that lipid diffusion dominates over any rotations in their paper. We made a careful analysis of the impact of rotation on the results from our model and found that while rotation does change runlength, force correlations, motor off rates etc, there is no qualitative changes in the results when compared between rigid and lipid cargoes and hence it doesn't impact any of the conclusions of our study.

More minor comments:

1. I did not like the authors stating that they had demonstrated various things "for the first time" — particularly given that they did not seem aware of some previous related work (see major comment 1).

We agree that this needs to be toned down as noted by Reviewer 1 as well. We have removed such statements.

2. I thought some of the claims in the abstract, particularly in the last two sentences, were not clearly backed up in the paper.

We have rewritten the second half of the abstract, so hopefully this should not be a problem any more. In particular we removed the reference to “mechanical efficiency” which we did not define and was therefore unclear.

3. In the author summary, I thought there should be a sentence explaining why motors are non-cooperative. Otherwise, it seems like the authors are saying that the motors are non-cooperative and then explaining why they are, indeed, cooperative (or at least not non-cooperative, if you will pardon a double negative).

It was somewhat hard to explain this within the word limit constraint of the author summary especially given the audience. We used the word “unsynchronized” instead which may give an intuitive sense of the problem for the non-expert audience of the author summary. If the reviewer has any suggestions for this, we are glad to take them.

4. Is “increased mechanical efficiency,” referenced at the end of the introduction, defined and demonstrated?

We have now removed this part since we do not specifically address it.

5. Throughout the paper, the word “significantly” seems to be used to refer to things that appear different (i.e., “This distribution … is significantly broader …” bottom of page 3). I would use a different word, so it is not conflated with statistical significance.

We appreciate this point. We have now taken care to not use the word significantly unless we mean statistical significance.

6. Equation 14, the exponential dependence of reaction rate on force, is often attributed to Bell (GI Bell, Science, 1978). The connection between that and Kramers theory was known prior to Schnitzer et al 2000 (e.g. Evans and Ritchie, Biophysical Journal, 1997).

We thank the reviewer for pointing this out. We have now cited GI Bell, Science, 1978 and Evans and Richie, 1997.

7. I found it confusing that the supplementary sections each had different reference numbering than the main text. Perhaps this is journal style and would be less confusing when the supplements are not in the same document as the main text; however, I lost some time looking through references from the main text instead of the appropriate reference from the supplement.

We apologize for this confusion. Yes, in Plos CB the SI is published separately from the main text. So it might indeed be less confusing then. But we understand that this is confusing when we put them together.

8. In appendix S3, it appears that reference 23 of the main text is cited, while all other references in the appendices are self-contained.

Thanks to the referee for pointing this out. We have resolved this issue.

9. Overall, I thought the figures might communicate information a little bit more efficiently. For example, the same symbols/colors might be used to indicate the amount of cargo fluidity throughout the paper, symbols might be defined more clearly (e.g. in Fig. 3d, one has to read through the text to find out what the deltas represent).

This is indeed a very good suggestion. We are now more consistent with our use of colors for lipid (greenish) and rigid cargo (brown). In fig 5c, we have now indicated the meaning of the different deltas on the figure itself to make it convenient for the reader.

10. It should be mentioned that the viscosity of water used in the paper applies to room temperature (~20 C).

Yes, we have now included this point.

Reviewer #3:

In this manuscript, the authors use Brownian dynamics simulations to explore transport by molecular motors that are attached to their cargo by fixed anchor points vs. by motors that can diffuse on a fluid cargo surface. Analyzing several complementary metrics such as force distributions and detachment rates of motors, they conclude that surface fluidity provides a simple mechanism to promote load sharing between individual motors, and thus leads to an increase in the number of active motors pulling on the cargo, as well as to increased run lengths of the cargo.

The study is well-performed and detailed, and the conclusions regarding the force sharing properties between motors are relevant and interesting for the general question of cargo transport by teams of motors. However, the manuscript in its current form provides a rather convoluted presentation of the main results, lacks relevant comparisons with experimental observations, and does not discuss comprehensively alternative scenarios that might influence the conclusions. I therefore think additional sets of simulations, a genuinely thorough restructuring of the text, and a corresponding

revision of the figures are necessary to consider this study for publication in PLoS Computational Biology.

Below I list my major concerns:

M1) In general, physical mechanisms that impact the mechanical interference between motors are not discussed carefully and in sufficient detail: Basic aspects of load sharing & mechanical coupling between bound motors should be elucidated with further analysis from individual motor trajectories. For instance, what are typical separations between bound motors? How do these separations translate into force generation profiles? What are the typical timescales of force balance between motors? In the "Conclusion" the authors say "..lipid membrane which allows the attachment points to move and relax strain…", **but in fact the effect of a fluid membrane should be more of a delay in strain generation rather than relaxation of strain**. Timescales of strain generation or force balance between bound motors for the two different cases of fluid vs rigid cargo could reveal further mechanisms underlying the changes e.g. in on/off rates or force generation profiles.

We agree with the reviewer that further examination of some of the details of the physical mechanisms underlying the mechanical interference would be good. We are thankful for the suggested analysis which has helped us gain further insights into the system. As suggested, we measured the typical separation between motor anchor positions and also head positions in rigid and lipid cargoes along with the corresponding motor forces. Please find the data in (Fig 2(a) and S1 Figure). It can be seen in Fig 2(b) that the motor anchor positions and head positions are more dispersed and also on the opposite sides of cargo in case of rigid cargo and on the same side in case of lipid cargo. The force correlation data of Fig 2(e) also supports this idea that motors are likely to be on opposite sides in rigid cargoes and on the same side in case of lipid cargoes. Motor forces (see S1 Figure) as a function of time doesn't show signs of slow strain buildup. It is seen that forces are random fluctuations in both rigid and lipid cargo, evidently rigid cargo having higher likelihood of higher force amplitudes compared to lipid cargo (inferring from the distribution of motor force amplitudes in Fig 2(b) and 2(c)).

M2) Different motor stiffness regimes needs to be explored. The stiffness value used in this work (k_{mot} = 0.32pN/nm) is taken from a rather old study (Refs [59] and [60]). However, much smaller stiffness values within the range 0.05-0.2pN/nm were used in more recent studies such as in Driver et al, PCCP 2010. These different stiffness regimes might markedly influence the off-rates or average run lengths of the cargo, e.g. as explored in Berger et. al, PRL.

We thank the reviewer for pointing this out and agree that it would be good to look at the robustness of our results with respect to motor stiffness . As suggested , we performed simulations for a range of stiffness values: 0.1 pN/nm - 0.5 pN/nm. Please find the data from these simulations below. The average motor off rate is observed to increase by almost 20% for a change in the motor stiffness from 0.1 pN/nm to 0.5 pN/nm for rigid cargo but much less for lipid cargo. Run length and average number of bound motors also decrease with an increase in the stiffness, consistent with the increase in motor off-rate. Please find the data below (Also added as in the supplement S22 Fig)

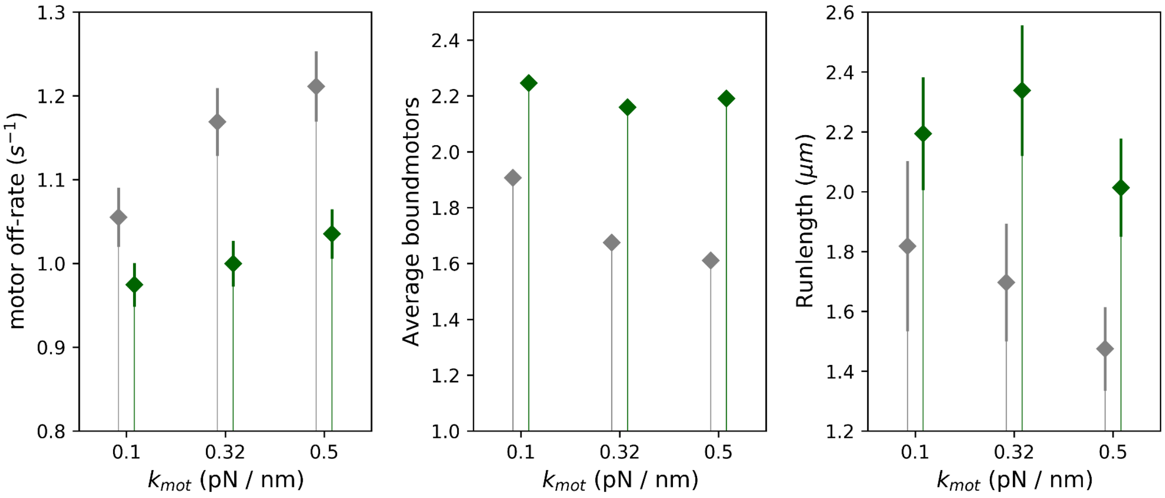

motor off rate, Average number of bound motors and the runlength for different values of motor stiffness. We used the same methods for measuring these metrics as described in the paper. N=16, [ATP] = 2mM.. Dark green - lipid cargo, gray - rigid cargo

M3) It is unclear to me why the authors only consider cargos of a single size (with radius R = 250 nm). This is an important aspect that would presumably influence the attachment of and interference dynamics between motors. How do the main results such as the force distributions (Fig.2b), on & off rates of motors (Figs. 2e&g), as well as the mean run lengths (Fig.3c) depend on the cargo size, such as explored in Erickson et al PLoS Comp Biol 2011?

We thank the reviewer for suggesting this analysis. Indeed, as the reviewer expected, the radius of the cargo influences the interference dynamics between the motors. Please find data of magnitude of motor forces, force correlations, etc. for different cargo radii below (Also added as S7 Figure). The distribution of forces (S7 Fig a and b) shows no clear trend except perhaps for a marginal increase in larger forces with decreasing radii for lipid cargo, which could be due to the steric constraints becoming more important. Interference between motors, quantified by the force correlations (S7 Fig c), show negligible correlations between motors for lipid cargo at any size, while for rigid cargo, the interference appears to increase with size, consistent with potentially increased spacing between motors. Motor off-rate (S7 Fig d) decreases as a function of cargo size

for both rigid and lipid cargo but the off-rate of motor in lipid cargo is lower than rigid cargo for all cargo sizes, which is again consistent with the picture of reduced interference and greater load-sharing with lipid cargo. Runlength values decrease exponentially with increase in radii for both rigid and lipid cargoes (S21 Fig) The difference in the runlength values between lipid and rigid cargo is highest for lowest cargo size and decreases with increase in size. This result is consistent with findings in Bovyn et. al. 2021 and our analytical estimates in (S18 Fig). Average number of motors also follows the same trend as the runlength. The runlength results for rigid cargo matches the results in Erickson et. al. 2011 (see Figure 15 and Figure 11, 1x- No rotation) In Figure 11, 1x- No rotation, we don't see much of a decrease in runlength presumably because in their case the cargo has only N=5 motors. In this low motor regime, we expect the cargo to be effectively hauled by a single motor, their runlength values seem to be constant 800 nm that corresponds to the runlength of a single motor. However, average number of motors decreases considerably with increase in radius in

Erickson et. al. 2011 (see Figure 15) for high number of motors on the cargo.

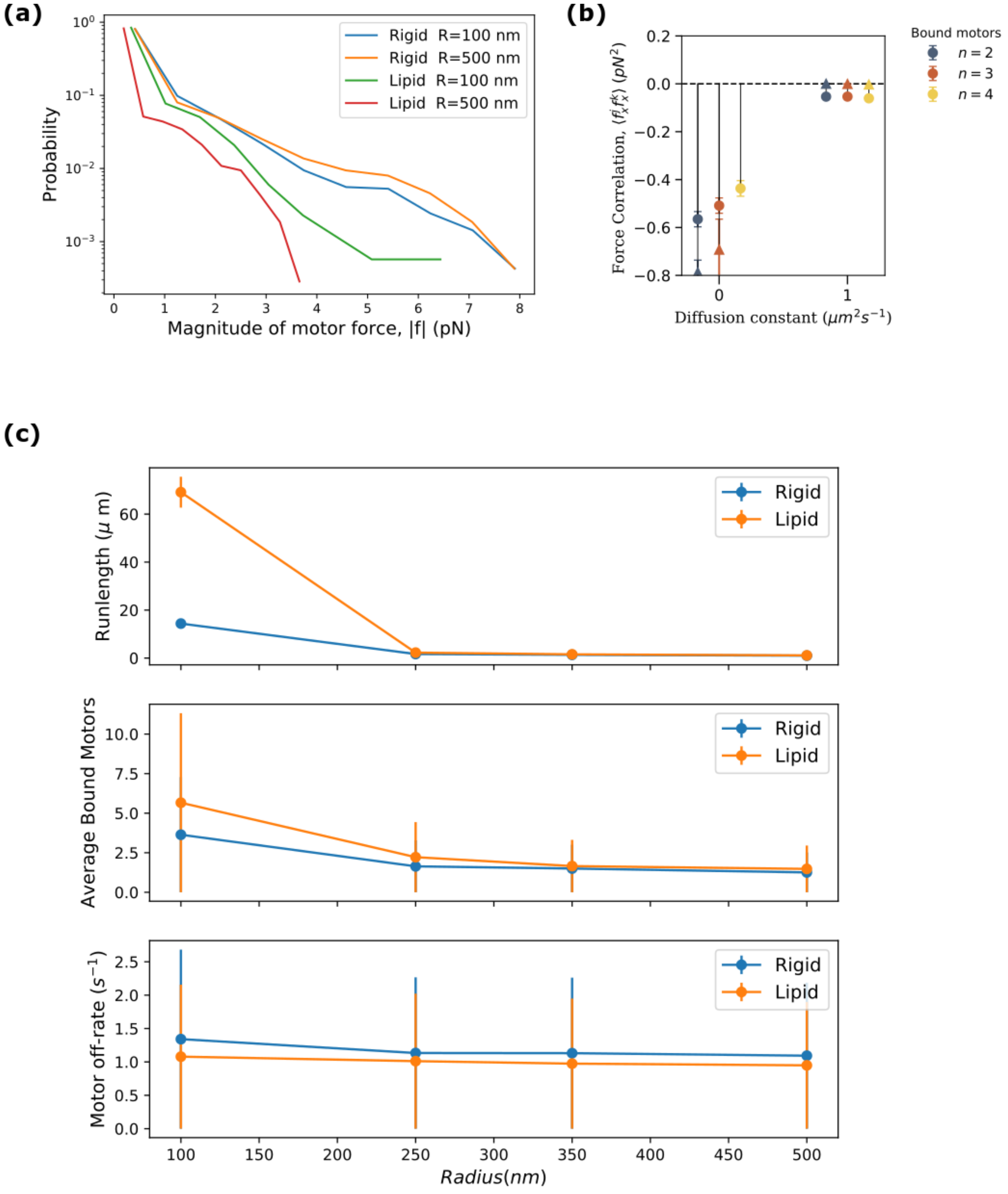

(a) Distribution of the absolute magnitude of motor forces in rigid and lipid cargoes for different cargo radii. **(b)** Force correlations between bound motors for different values of cargo radii. (Radius Values: Circles =100 nm, Triangles = 500 nm) **(c)** Runlength, average number of bound motors and average off-rate of motor in lipid and rigid cargies as a function of the cargo radius. N=16, [ATP]= 2mM, simulations without rotation.

M4) Similar to M3, how would different parameter regimes of the motors influence the main results? This could be relevant to gain insights on distinct motor types such as myosins or other kinesins. For instance, would the load sharing properties change significantly if the motors had a small “no-load velocity” v_0 or a small stall force F_s? How would these different parameter sets influence the number of and the forces experienced by bound motors, such as explored in Ucar & Lipowsky, Nano Lett 2020?

We thank the reviewer for this suggestion and agreed that a study of collective force generation under load for varying motor speed and stall force would be a nice addition. We ran simulations of cargo in a harmonic trap with (force constant 0.06 pN/nm) and measured the mean value of the x-component of the total force on cargo by the bound motors as was done in Ucar & Lipowsky, Nano Lett 2020. We measured the same rescaled collective force as measured in Ucar et. a. 2020. Our results show that collective force is highest for low stall force (Fs) and high unloaded motor velocity (v_0) for both rigid and lipid cargoes. This is in agreement with Ucar et. a. 2020. Additionally we see that the motors in lipid cargoes generate higher collective force compared to motors in rigid cargo. This analysis provides another direct evidence that the lipid membrane improves load sharing properties of teams of motors. *This is now a whole new subsection “Motor teams in fluid cargo generate higher collective forces” along with a new Fig.7*. It would be interesting to explore other single motor parameters and more quantitatively measure the changes in load sharing properties for different diffusion constants. We are planning to pursue that in our future work.

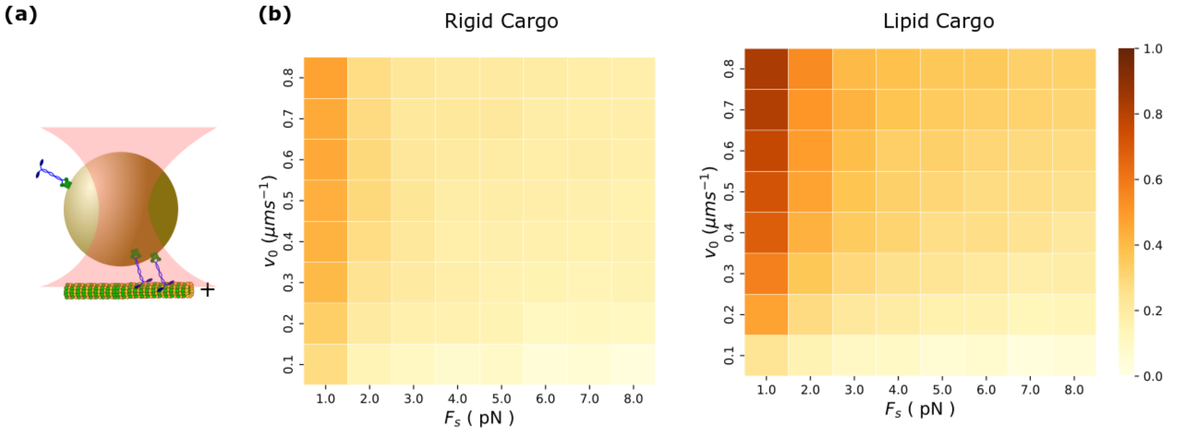

(a) Schematic of the optical trap set up that we used in simulation. (b) Rescaled mean collective force <Fx>/NFs (ratio of average value of the total x-component of forces exerted by the team of motors on the cargo divided by NxFs) unloaded motor velocity (vo) and stall force of motor (Fs) in rigid and lipid cargoes.

200 cargo runs were used for each parameter set. For each cargo run, data of the number of bound motors and motor forces were recorded at a sampling rate of 100/s for maximum time of 1 s for each cargo run. Mean collective force is calculated as the average of the total x-component of motor forces over all time samples and cargo runs. N=4 and [ATP] = 2mM, rotational diffusion was considered. Lipid Cargo diffusion constant is 1 um^2/s.

M5) In general, the authors should try to include some experimental data to quantitatively test / compare their theoretical results. Otherwise their claim, in the abstract, "...th[u]s allowing us to reconcile different experimental results in different regimes" remains unsubstantiated. For example, it should be further elucidated with more targeted analysis and additional simulations / datasets from the literature why cargo velocities (Fig. S14) do not change significantly in contrast with previous experimental findings such as in Grover et al PNAS 2016.

We appreciate this suggestion and have now attempted to add more connections to experimental results throughout. For example, we have now addressed why cargo velocity doesn't change in the *Conclusion*. Now we have added to this section a short discussion on the discrepancy between our velocity results and Grover et. al. PNAS 2016.

Technical / minor comments:

m1) Fig. 3 is a rather arbitrary collection of different metrics, and it is very tiresome to extract a significant message out of it. In general, each item (i.e. Figure) in the manuscript should convey a single message. It might therefore be beneficial to include captions as single sentences for each figure.

We agree. To make it easier to parse the information, we have split this figure into two separate figures (Figure 4 and Figure 5). Figure 4 presents data on enhanced motor accumulation in the lipid cargo. Figure 5 is now focused on the enhanced run length of lipid cargo under suitable parameter regimes and analytical estimates of the contribution to lipid cargo runlength from different effects. We have also added a description of the different symbols (Delta) in Figure 5(c) to make it easy to take away the main idea without having to go through the entire discussion.

m2) Fig.3(b): A schematic for the polar angle \theta is necessary. Top panel: it is not very informative to plot constant probability values over a large set of \theta values because the cargo rotations are neglected in simulations. Bottom panel: The message is presumably that the probability of motors accumulating at the bottom of the cargo

increases over time but the plotting is very confusing. It might be better to complement these angles with distributions of anchor-anchor distances.

We appreciate these suggestions and have now added a schematic for the polar angle. We have also measured the distribution of anchor-anchor distances (see S12 Fig). Overall the distributions are not too different, but rotations seem to allow for larger separations in rigid cargo.

m3) Fig.3(d): This plot as well as the corresponding section (Fluid cargo have longer tun lengths than rigid cargo) in the main text are extremely tedious to read due to the many different quantities introduced in passing and it remains again unclear which message should be extracted from the different analytical estimates. This entire section and Fig.3, in my perspective, should be largely revised and rethought to clearly explain the important conclusions.

We agree and we have therefore broken down this long section into several subsections, each with a clear topic and message. We hope this has improved the clarity.

m4) Details are missing on how the explored metrics (such as forces acting on individual motors and force correlations) are calculated.

We have written a separate appendix (S5 appendix) with a schematic diagram and explanations. In addition to that we have added a schematic in main text (Figure 1c) which indicates how we compute motor forces and sign convention for the magnitude of motor force in hindering and assisting configurations.

m5) In conclusion, the authors state "rigidly coupled motors experience…", however, the motors are elastically coupled even in the case of a rigid cargo due to their elastic linkers. The terminology overall should be used more carefully.

In conclusion, instead of phrase "rigidly coupled motors" we used phrase "motors coupled through a rigid cargo"

Have the authors made all data and (if applicable) computational code underlying the findings in their manuscript fully available?

The PLOS Data policy requires authors to make all data and code underlying the findings described in their manuscript fully available without restriction, with rare exception (please refer to the Data Availability Statement in the manuscript PDF file). The data and code should be provided as part of the manuscript or its supporting information, or deposited to a public repository. For example, in addition to summary statistics, the data points behind means, medians and variance measures should be available. If there are restrictions on publicly sharing data or code —e.g. participant privacy or use of data from a third party—those must be specified.

Reviewer #1: Yes

Reviewer #2: Yes

Reviewer #3: **No:** I cannot find a link to a public repository where the simulation code can be found.

The simulation code and data in main text can be found in the following github repository: https://github.com/nsarpangala/lipid-cargo-transport
