# Supplementary material for: Cargo surface fluidity can reduce inter-motor mechanical interference, promote load-sharing and enhance processivity in teams of molecular motors
Source: PLoS Comput Biol. 2022 Jun 8;18(6):e1010217. doi: 10.1371/journal.pcbi.1010217 (PMC9212169; doi:10.1371/journal.pcbi.1010217)
Supplement: Attachment — Submitted filename: PLOS CB Response 2.pdf [file pcbi.1010217.s033.pdf]

We would once again like to express our appreciation for the very careful and thorough reading of the manuscript by the reviewers and the very helpful and detailed constructive criticisms and suggestions in the previous round. Based on the comments and suggestions, we made major changes to the paper that tremendously improved the quality, readability and potential impact and we are glad that the referees are overall satisfied with the changes. We have acknowledged their efforts in the paper as a token of our appreciation.
Below, we address the reviewer's few remaining comments (in blue) point by point. We note that changes to the manuscript are indicated in blue text in the main paper draft.

Reviewer #1: The authors have done all that is reasonable to address my comments and comments of other reviewers. I am therefore in support of publication.
We thank the reviewer once again for all their efforts!

Reviewer #2: The authors have addressed most of my concerns except for:

1. (Line 337) The source of negative cooperativity in the model of Lombardo et al. 2017, according to my understanding of the paper, is not competition for binding sites. Instead, it is due to myosin binding restricting the motion of the cargo and thereby making it more energetically costly for subsequent motors to bind.
2. Along the same lines, actin is not a linear track (as stated a couple times in the paper, e.g. line 336, line 745). It has two protofilaments arranged in a helix.
We agree that it is indeed the steric effect mentioned by the referee coupled with the incompressibility of the motors that leads to negative cooperativity in Lombardi. We only meant to highlight that this effect is much less significant for kinesins as there are many more protofilaments that are geometrically accessible to the unbound kinesins on a microtubule than for myosins on an actin filament, which as the referee correctly points out, has two protofilaments. We have amended our wording in the main text and in the methods appropriately.

3. While I appreciate the difficulty in writing a pithy and understandable significance statement, I do not think "unsynchronized" is the right word. It seems to me that the authors are trying to address the issue that processive motors work as a team in vivo, yet seem to work poorly as a team when coupled rigidly in vitro. They are adding to a growing body of literature that argues that membrane fluidity resolves this contradiction.
We thank the referee for this suggestion and have appropriately modified the author summary.

4. (Line 365) A reference to a Table is broken.
Thanks for catching this. This has been fixed.

Reviewer #3: The substantial revisions of the authors have improved the manuscript to provide a more thorough analysis and a more balanced presentation and discussion of their modelling and results. I therefore recommend publication in general. However, I think some sections of the manuscript could benefit from improving the writing to increase the overall accessibility and thus eventual impact of the paper. This is most evident in the abstract, which is rather long and not quite eloquently written. Some suggestions (not to be taken literally but as guidelines) are:

(i) removing unncessary/uninformative words (e.g "simulation" instead of "computer simulation", removing "canonically non-cooperative").
(ii) replacing technical terms with more accesible alternatives (e.g. "..show that surface fluidity enhances mechanical load sharing between kinesins, which increases the average duration of

motors on the filament and thus their collective processivity.” instead of “surface fluidity could lead to the reduction of negative mechanical interference between kinesins, enhancing load sharing thereby
decreasing single motor off-rates and increasing processivity.”)
(iii) replacing informal expressions (e.g. “transport distances” instead of “travel distances”)
We thank the reviewer for these suggestions and have taken to the opportunity to shorten and simplify the abstract as appropriate.

Have the authors made all data and (if applicable) computational code underlying the findings in their manuscript fully available?
The PLOS Data policy requires authors to make all data and code underlying the findings described in their manuscript fully available without restriction, with rare exception (please refer to the Data Availability Statement in the manuscript PDF file). The data and code should be provided as part of the manuscript or its supporting information, or deposited to a public repository. For example, in addition to summary statistics, the data points behind means, medians and variance measures should be available. If there are restrictions on publicly sharing data or code —e.g. participant privacy or use of data from a third party—those must be specified.
Reviewer #1: Yes
Reviewer #2: Yes
Reviewer #3: Yes
